# Splice variants as novel targets in pancreatic ductal adenocarcinoma

**DOI:** 10.1038/s41598-017-03354-z

**Published:** 2017-06-07

**Authors:** Jun Wang, Laurent Dumartin, Andrea Mafficini, Pinar Ulug, Ajanthah Sangaralingam, Namaa Audi Alamiry, Tomasz P. Radon, Roberto Salvia, Rita T. Lawlor, Nicholas R. Lemoine, Aldo Scarpa, Claude Chelala, Tatjana Crnogorac-Jurcevic

**Affiliations:** 1 Centre for Molecular Oncology, Barts Cancer Institute, Queen Mary University of London, John Vane Science Centre, London, EC1M 6BQ UK; 2 ARC-Net Research Centre and Department of Diagnostics and Publich Health, Section of Pathology, University and Hospital Trust of Verona, Verona, Italy

## Abstract

Despite a wealth of genomic information, a comprehensive alternative splicing (AS) analysis of pancreatic ductal adenocarcinoma (PDAC) has not been performed yet. In the present study, we assessed whole exome-based transcriptome and AS profiles of 43 pancreas tissues using Affymetrix exon array. The AS analysis of PDAC indicated on average two AS probe-sets (ranging from 1–28) in 1,354 significantly identified protein-coding genes, with skipped exon and alternative first exon being the most frequently utilised. In addition to overrepresented extracellular matrix (ECM)-receptor interaction and focal adhesion that were also seen in transcriptome differential expression (DE) analysis, Fc gamma receptor-mediated phagocytosis and axon guidance AS genes were also highly represented. Of note, the highest numbers of AS probe-sets were found in collagen genes, which encode the characteristically abundant stroma seen in PDAC. We also describe a set of 37 ‘hypersensitive’ genes which were frequently targeted by somatic mutations, copy number alterations, DE and AS, indicating their propensity for multidimensional regulation. We provide the most comprehensive overview of the AS landscape in PDAC with underlying changes in the spliceosomal machinery. We also collate a set of AS and DE genes encoding cell surface proteins, which present promising diagnostic and therapeutic targets in PDAC.

## Introduction

Alternative splicing (AS) is one of the key regulatory events leading to transcriptome and proteome diversity. From bacteria and archaea, AS has, through increased prevalence, contributed to driving speciation and shaped the evolution of all multicellular organisms, including our own species^1^. Multiple distinct transcript variants are encoded by the majority, if not all human multi-exon genes, and more than half of all AS events show tissue specificity, thus highlighting a critical role of AS in creating phenotypic complexity^2–4^.

The functional consequences of most AS events are still unknown, with some resulting in non-functional products, some in the splice variant product(s) that assume completely different function from the wild-type protein, and in some cases the proteins even acquire antagonistic functions (for review see ref. 5). It is not surprising then that cancer has harnessed this important regulatory mechanism, which is implicated in all the ‘hallmark’ pathways of cancer^6^, and can hence be considered an additional cancer hallmark itself^7^. Some of the spliced variants in pre-malignant lesions have also been found in advanced cancers, suggesting their potential as drivers of cancer development and progression^8^.

Distinct patterns of cancer-specific splicing when compared to normal tissue counterparts have been reported for a variety of tumours^9, 10^, some of which could play a role as promising diagnostic, prognostic and/or therapeutic targets^5^. In pancreatic ductal adenocarcinoma (PDAC), in contrast to a wealth of other genomic data, except for a cell line study^11^, no AS data on a whole genome scale have yet been reported. A detailed understanding of the alternative splicing landscape in PDAC is therefore warranted; with this in mind, we have undertaken an in-depth investigation of AS events combined with whole transcriptome analysis, and explored the alterations in the splicing machinery itself in normal and pancreatic cancer specimens.

## Results

### Exon-based whole transcriptome profiling in PDAC

We used Affymetrix exon array to generate whole exome-based profiles for 28 PDAC (Table 1) and six normal pancreas bulk tissues. Although widely used as a normal comparator, we are aware that this can be a potential source of bias when comparing non-ductal acinar tissue versus ductal-differentiated carcinoma.

**Table 1:** Demographic and clinical information of the analysed PDAC samples.

PDAC case number	Gender	Age	Grade	pT	pN	pM	Analysis
13	F	73	G2	T3	N1	M0	EA
14	M	64	G2	T3	N1	M0	EA
16	F	50	G2	T3	N1	M0	EA/qPCR
17	F	59	G3	T3	N1	M0	EA/qPCR
19	M	62	G2	T3	N1	M0	EA/qPCR
20^*^	M	63	G3	T3	N1	M0	EA
21	F	77	G2	T3	N1	M0	EA
22	M	80	G2	T3	N1	M0	EA/qPCR
23	M	73	G2	T3	N1	M0	EA/qPCR
24	F	59	G3	T3	N1	M0	EA/qPCR
25	F	71	G2	T3	N1	M0	EA
27	F	42	G2	T2	N1	M0	EA/qPCR
32	M	67	G2	T3	N1	M0	EA/qPCR
35	F	68	G2	T4	N0	M0	EA
46	M	68	G2	T1	N0	M1	EA/qPCR
54	F	41	G3	T2	N0	M0	EA/qPCR
55	M	55	G2	T3	N1	M0	EA
60	F	65	G3	T3	N1	M0	EA/qPCR
63	M	67	G3	T4	N1	M0	EA/qPCR
64	M	59	G3	T3	N1	M0	EA
80	M	74	G2	T3	N1	M0	EA
100	F	73	G2	T3	N0	M0	EA/qPCR
102	F	73	G2	T3	N1	M0	EA
108	M	59	G3	T4	N1	M0	EA
150	M	53	G3	T3	N1	M0	EA
163	M	64	G2	T3	N1	M0	EA
169	M	66	G2	T4	N1	M0	EA
173	M	60	G3	T3	N1	M0	EA
89	M	81	G2	T4	N1	M0	qPCR
71	F	67	G3	T3	N1	M0	qPCR
73	F	74	G2	T3	N1	M0	qPCR
75	F	32	G2	T3	N0	M0	qPCR
95	M	68	G3	T3	N1	M0	qPCR
152	M	62	G3	T3	N0	M0	qPCR
166	F	65	G2	T3	N1	M0	qPCR
11	F	59	G3	T3	N1	M0	qPCR
15	F	67	G3	T3	N1	M0	qPCR

^*^This sample was derived from the patient that received neoadjuvant therapy (Gemcitabine + Oxaliplatin; all the remaining samples were obtained from naive patients. EA = exon array; qPCR = quantitative polymerase chain reaction; pTNM (pathology staging: Tumour, lymph Nodes, Metastasis).

**Figure 1:**
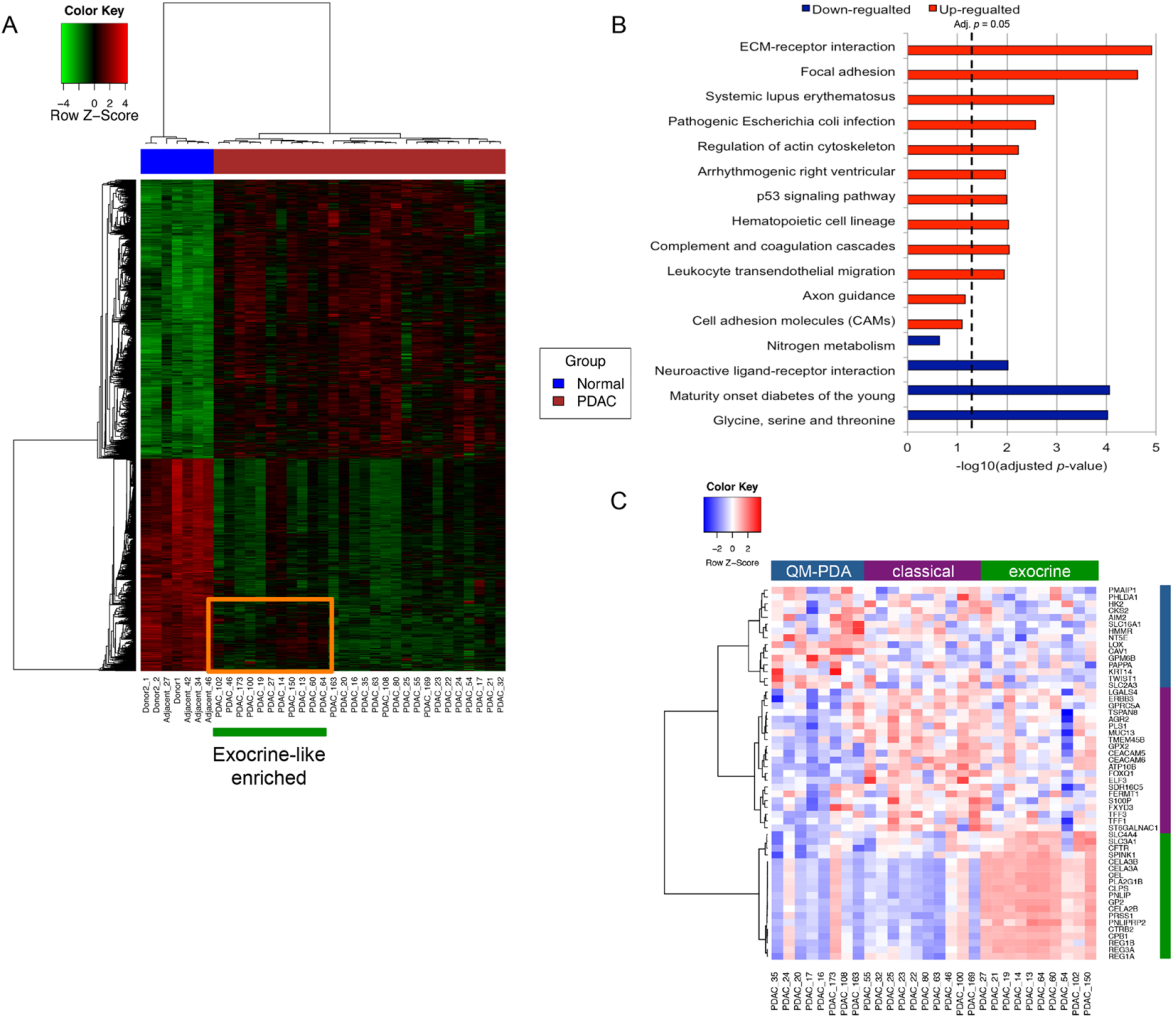
Differentially expressed protein-coding genes in PDAC. (**A**) Heatmap of the 1,887 differentially expressed transcript clusters in PDAC. The heatmap represents relative over- (red) and under-expressed (green) genes. The covariates at the top represent tumour (red) and normal (blue) samples. A subgroup of 11 PDAC samples is indicated by the orange box, determined by the exocrine-like gene signature by Collisson *et al*. with higher expression in a cluster of 255 transcripts than other PDAC samples. (**B**) KEGG pathway enrichment test for significantly up- and down-regulated genes in PDAC. The BH adjusted *p*-values were used (x-axis). (**C**) Heatmap and sample clustering based on the Collisson signature dividing samples into three molecular subtypes.

Firstly, we analysed the expression profiles for 225,925 probe-sets across all exons and 17,528 transcript clusters after intensity filtering for all samples (see Methods). Unsupervised hierarchical clustering demonstrated a clear separation of the control and PDAC sample groups based on both probe-set and transcript level expression (Supplementary Fig. S1). In total, 1,887 transcript clusters were differentially expressed (DE) in PDAC compared to normal pancreas, of which 1071 (56.8%) were upregulated and 816 (43.2%) downregulated. Among these, 1,600 corresponded to protein-coding genes, with 917 (57.3%) upregulated and 683 (42.7%) downregulated transcript clusters in PDAC (Fig. 1A, Supplementary Table S1A). The KEGG pathway enrichment test using DAVID^12^ suggested that the identified overexpressed genes in PDAC were highly enriched for extracellular matrix (ECM)-receptor interaction (adj. *p* = 1.22e-05) and focal adhesion (adj. *p* = 2.37e-05), while for downregulated genes in PDAC, glycine, serine and threonine metabolism (adj. *p = *9.40e-05) and maturity onset diabetes of the young (adj. *p* = 8.75e-05) were highly overrepresented (Fig. 1B, Supplementary Table S1B). The top deregulated canonical pathways based on Ingenuity pathways analysis (IPA) included small GTPases (CDC42, Rho), integrin and actin cytoskeleton signalling, again pointing to ECM-interaction, adhesion and cytoskeletal regulation. A full list of the pathways with the corresponding key genes is provided in Supplementary Table S1C. Analysis of the obtained differential expression profiles using the Pancreatic Expression Database^13^ revealed that out of 917 upregulated and 683 downregulated protein-coding genes in PDAC, 657 (71%) and 219 (32%) were previously reported displaying the same directions of changes, respectively (Supplementary Table S1D **)**. Among the newly discovered deregulated genes (701/1600; 44%), IPA revealed ephrin receptor and G-protein regulatory Gαi signalling as top deregulated canonical pathways (Supplementary Table S1E). Therefore, our exon-based expression analysis, with over four-fold increase in probe density in comparison to the ‘classical’ 3′-based arrays, provided a far more accurate and comprehensive portrait of pancreatic cancer transcriptome.

Interestingly, the transcriptomic profile of the 28 analysed PDAC samples also indicated the existence of two PDAC subgroups (Fig. 1A). This has prompted us to interpret our analysis in light of the previously reported PDAC subclassification by Collisson *et al*.^14^; the three PDAC groups: exocrine-like (n = 10), classical (n = 10) and QM-PDA (n = 8) are shown on Fig. 1C. The 11 closely grouped PDAC samples based on all 1,887 DE transcript clusters as indicated by the orange block in Fig. 1A were highly enriched for exocrine-like samples (n = 8, 73%). This group showed a higher level of expression of 255 transcripts compared to the rest of the PDAC samples, including pancreatic lipase-related protein *PNLIPRP2* and chymotrypsin-like elastase family, member 2B (*CELA2B*) genes, classical acinar-originating genes (Supplementary Table S1F). KEGG pathway analysis further showed, in contrast to the other, ‘non-exocrine’ PDAC subgroup, that this cluster of genes was also highly enriched for glycine, serine and threonine metabolism, glutathione metabolism and selenoamino acid metabolism as well as Maturity onset diabetes of the young (Supplementary Table S1F), pointing to potential underlying metabolic and functional differences between the two subgroups. However, no difference in survival between exocrine-like enriched and ‘non-exocrine’ patients’ groups was seen (log rank *P* = 0.822). Furthermore, no significant difference in survival was present between any of the three Collisson subgroups shown in Fig. 1C (log rank *P* = 0.277).

After integration of our data with the data from Zhang *et al*. (n = 70 in the merged dataset)^15^ (Supplementary Fig. S2), no significant association of any of the three PDAC subgroups with survival was identified. This is in agreement with the study by Moffitt *et al*.^16^, where the sample grouping was determined by the histology (as similarly confirmed recently by Bailey *et al*.^17^) with the presence of the stable exocrine-like signature in PDAC reflecting the remnant exocrine-like compartment in the samples. Therefore, our results are not inconsistent with the cited studies^16, 17^, and have to be interpreted in light of the interrogated material, which in large part ( > 60%) comprised the malignant cell component.

### Alternative splicing landscape in pancreatic cancer

**Figure 2:**
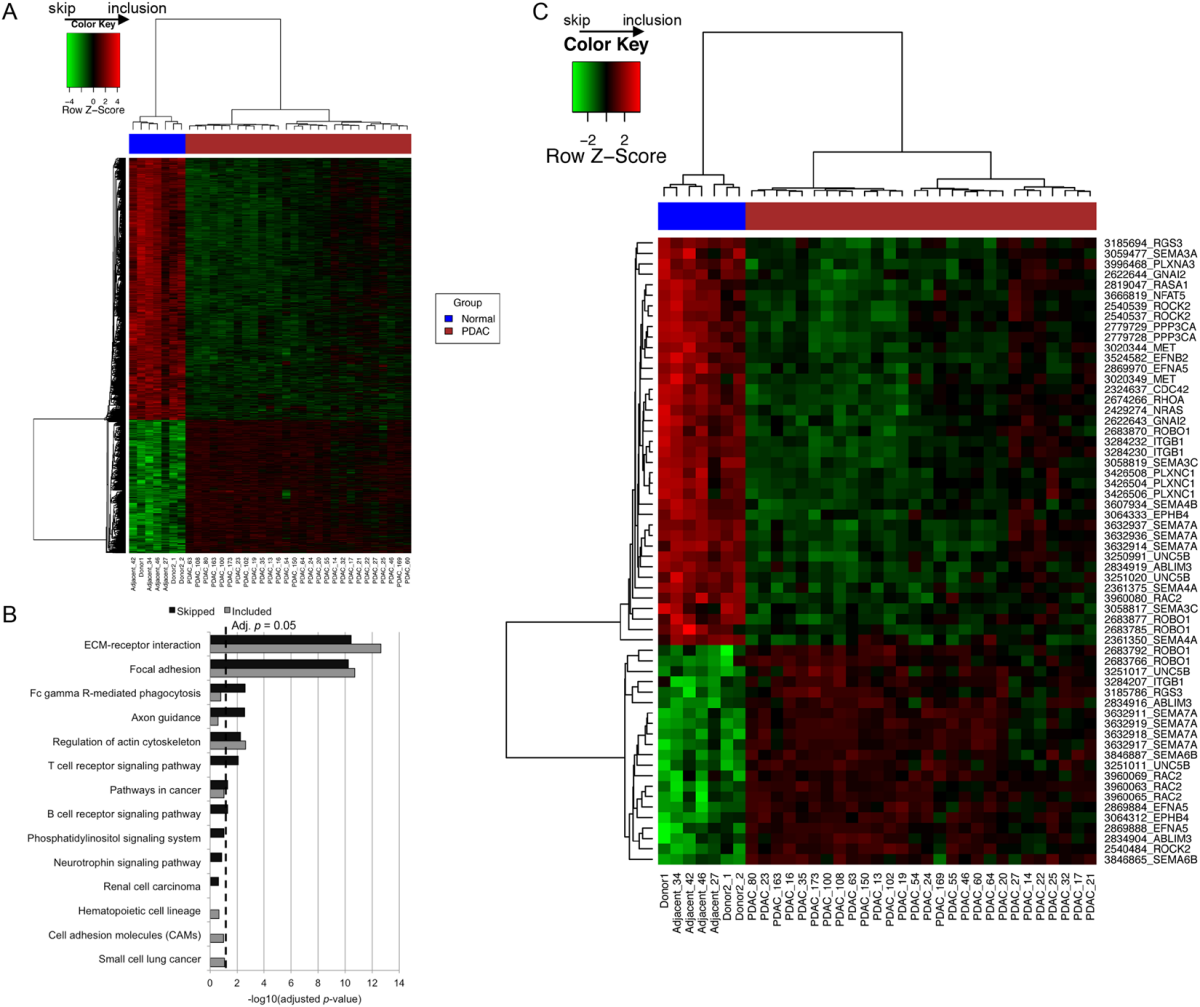
Alternative splicing in PDAC. (**A**) Heatmap of the 2,816 alternatively spliced probe-sets based on the FIRMA scores, with the relative inclusion shown in red and skipping shown in green. (**B**) KEGG pathway enrichment test for genes with included and skipped events. (**C**) Heatmap of 60 alternatively spliced probe-sets, representing 26 genes involved in axon guidance (KEGG). The row names are shown in the format of “probe-set” then followed by “gene symbol”.

After transcriptome analysis, we have undertaken a detailed AS analysis: in total, 2,816 differentially spliced probe-sets, representing 1,354 protein-coding genes, were identified (Supplementary Table S2A). This corresponded to on average 2.08 AS probe-sets per gene, ranging from 1–28. Of note, around half (1,424/2816; 50.5%) of these splicing events have not been reported previously in the Ensembl alternative splicing event set. The major AS event types found are listed in Table 2. Cassette exon (skipped exon, 14.3%) and alternative first exon (14.0%) were the most frequent, followed by intron retention (8.4%). Hierarchical clustering based on the FIRMA scores clearly separated the PDAC and control groups (Fig. 2A), suggesting a strong PDAC-specificity of the obtained splicing pattern. Interestingly, two subgroups of PDAC specimens were clearly evident. Since AS is shown to be associated with subtypes of breast cancer^18^, we explored this further, but subgrouping did not appear to correspond either to the one generated by DE signatures (Fig. 1A), nor any of the three Collisson sub-groups, and no difference in survival between the two groups was seen (log rank *P* = 0.433). We also did not find any association between tumour cellularity and tumour sample grouping based on DE or AS signatures. In addition, no association was observed between the tumour grade and these groups. However, it still remains to be explored if any other clinical correlates would emerge from these two AS subgroups in other studies.

**Table 2:** Alternative splicing event types in PDAC.

Splicing type	Number	Percentage (%)
Alternative 5′ sites (A5SS)	47	1.53
Alternative 3′ sites (A3SS)	43	1.67
Alternative first exon (AFE)	394	13.99
Alternative last exon (ALE)	33	1.17
Cassette exon (Skipped exon, CE)	402	14.28
Constitutive exon (CNE)	208	7.39
Intron retention (IR)	237	8.42
Intron isoform (II)	20	0.71
Mutually exclusive exons (MXE)	9	0.32

CE: an exon may be spliced out of the primary transcript or retained; CNE: constitutively spliced exons. All events were based on the nomenclature described in Wang *et al*.^4^.

Among differentially spliced probe-sets, 949 (33.7%) representing 555 genes were included in PDAC samples, while the remaining 66% (1,867 probe-sets/1,155 genes) were skipped in PDAC compared to normal samples (Supplementary Table S2A). Among 1,354 protein-coding genes with AS signatures, around half (638, 47%) had more than one alternatively spliced probe-set, while genes from the collagen family had the highest number of AS probe-sets, including 28 for *COL5A1*, 19 for *COL11A1*, 17 for *COL5A2*, 12 for *COL16A1* and 11 for *COL1A1* and *COL17A1* (Supplementary Table S2B). For example, AS events of *COL1A1* indicated by the 11 significant probe-sets in PDAC included skipped first exon (n = 1 probe-set) and highly included last exon/3′ UTR (n = 4), as well as cassette exons in the gene body (Supplementary Fig. S3A). These are significant findings, considering the abundance and increasingly recognised role of the stromal environment in PDAC.

In general, there was a weak positive correlation between the number of AS probe-sets and the number of total core probe-sets (Spearman’s rank correlation *r* = 0.26) and the number of exons (Spearman’s correlation *r* = 0.16), as noted previously for AS genes^3^, suggesting that selection pressure may play a role in limiting the splicing complexity in exceptionally large genes in PDAC as well.

The top ten most differentially spliced probe-sets (both skipped and included) are shown in Table 3.

**Table 3:** Top differentially spliced protein-coding genes between PDAC and normal pancreas.

Probe-set_ID	Transcript cluster_ID	Symbol	Definition	logFC (FIRMA)	Event	adj. P-Value
3824875	3824874	IFI30	Interferon, gamma-inducible protein 30	−11.93	skipped	6.42E-09
3861578	3861557	LGALS4	Lectin, galactoside-binding, soluble, 4	−10.80	skipped	2.66E-08
2829950	2829947	TGFBI	Transforming growth factor, beta-induced, 68kDa	−10.23	skipped	4.89E-09
2880347	2880292	DPYSL3	Dihydropyrimidinase-like 3	−9.75	skipped	5.24E-14
3632030	3631964	PKM2	Pyruvate kinase, muscle	−9.71	skipped	9.00E-11
3119348	3119339	LY6E	Lymphocyte antigen 6 complex, locus E	-9.57	skipped	4.96E-11
3405602	3405587	GPRC5A	G protein-coupled receptor, family C, group 5, member A	−9.54	skipped	2.14E-15
2598263	2598261	FN1	Fibronectin 1	−9.38	skipped	5.71E-08
3264344	3264326	ACSL5	Acyl-CoA synthetase long-chain family member 5	−9.28	skipped	4.21E-15
3058819	3058759	SEMA3C	Semaphorin-3C	−9.22	skipped	3.30E-11
3762200	3762198	COL1A1	Collagen, type I, alpha 1	13.64	included	3.86E-14
3013163	3013054	COL1A2	Collagen, type I, alpha 2	9.50	included	4.50E-11
3013162	3013054	COL1A2	Collagen, type I, alpha 2	8.44	included	7.09E-11
3762202	3762198	COL1A1	Collagen, type I, alpha 1	6.87	included	3.35E-13
3762203	3762198	COL1A1	Collagen, type I, alpha 1	6.35	included	4.02E-08
2535909	2535859	CAPN10	Calpain 10	6.30	included	7.07E-09
3351230	3351200	TMPRSS4	Transmembrane protease, serine 4	5.89	included	1.38E-09
3193625	3193482	COL5A1	Collagen, type V, alpha 1	5.81	included	9.30E-12
2405001	2404999	MARCKSL1	MARCKS-like 1	5.74	included	1.10E-11
2535912	2535859	CAPN10	Calpain 10	5.62	included	8.34E-07

To understand the putative roles of AS in PDAC, we performed detailed pathway analyses using both DAVID KEGG pathway and IPA enrichment tests. Interestingly, the KEGG pathway enrichment test suggested that AS genes were, similarly to DE genes, highly enriched for ECM-receptor interaction and focal adhesion (Fig. 2B; Supplementary Table S2C), highlighting again the importance of cancer cell-stroma interaction as a key biological feature of pancreatic cancer. In addition, Fc gamma R-mediated phagocytosis (adj. *p* = 2.49e-03) and axon guidance (adj. *p* = 2.81e-03) were also significantly overrepresented for genes with skipped probe-sets. Within the axon guidance pathway, the importance of which was recently flagged^19^, 26 genes (60 probe-sets) displayed significant AS signatures (Fig. 2C). While 10 of these genes (*ABLIM3*, *EFNA5*, *EPHB4*, *ITGB1*, *RAC2*, *RGS3*, *ROBO1*, *ROCK2*, *SEMA7A* and *UNC5B*) had both included and skipped probe-sets in PDAC, the majority (65%) of 60 AS probe-sets were skipped in PDAC (Fig. 2C; Supplementary Table S2D). For *ITGB1*, events of alternative (skipped) first exon, included and skipped exons and potential included last exon were identified. It is likely the short isoform was the preferred transcript in PDAC (Supplementary Fig. S3B **)**. The top AS signalling pathways in the Ingenuity pathway enrichment analysis also included integrin and axonal guidance signalling; a full list of the IPA pathways and key genes is provided in Supplementary Table S2E.

Among the 1,354 genes with AS signatures, 369 (27.3%) were genes coding for surface proteins (Supplementary Table S2F **)**; not surprisingly, these genes appeared to be highly enriched for cell adhesion (adj. *p* = 1.10e-22), cell surface receptor-linked signal transduction (adj. *p* = 1.60e-09), ECM-receptor interaction (adj. *p* = 3.7E-09), positive regulation of cell activation and proliferation (adj. *p* = 6.71e-07 and 9.42e-07, respectively), and the integrin-mediated signalling pathway (adj. *p* = 3.10e-06), comprising a number of integrins (I*TGA11*, *ITGA3*, *ITGA4*, *ITGA5*, *ITGAV*, *ITGB1*, *ITGB2*, *ITGB4* and *ITGB7* (Supplementary Table S2G).

### Real-time PCR validation of alternative splicing

**Figure 3:**
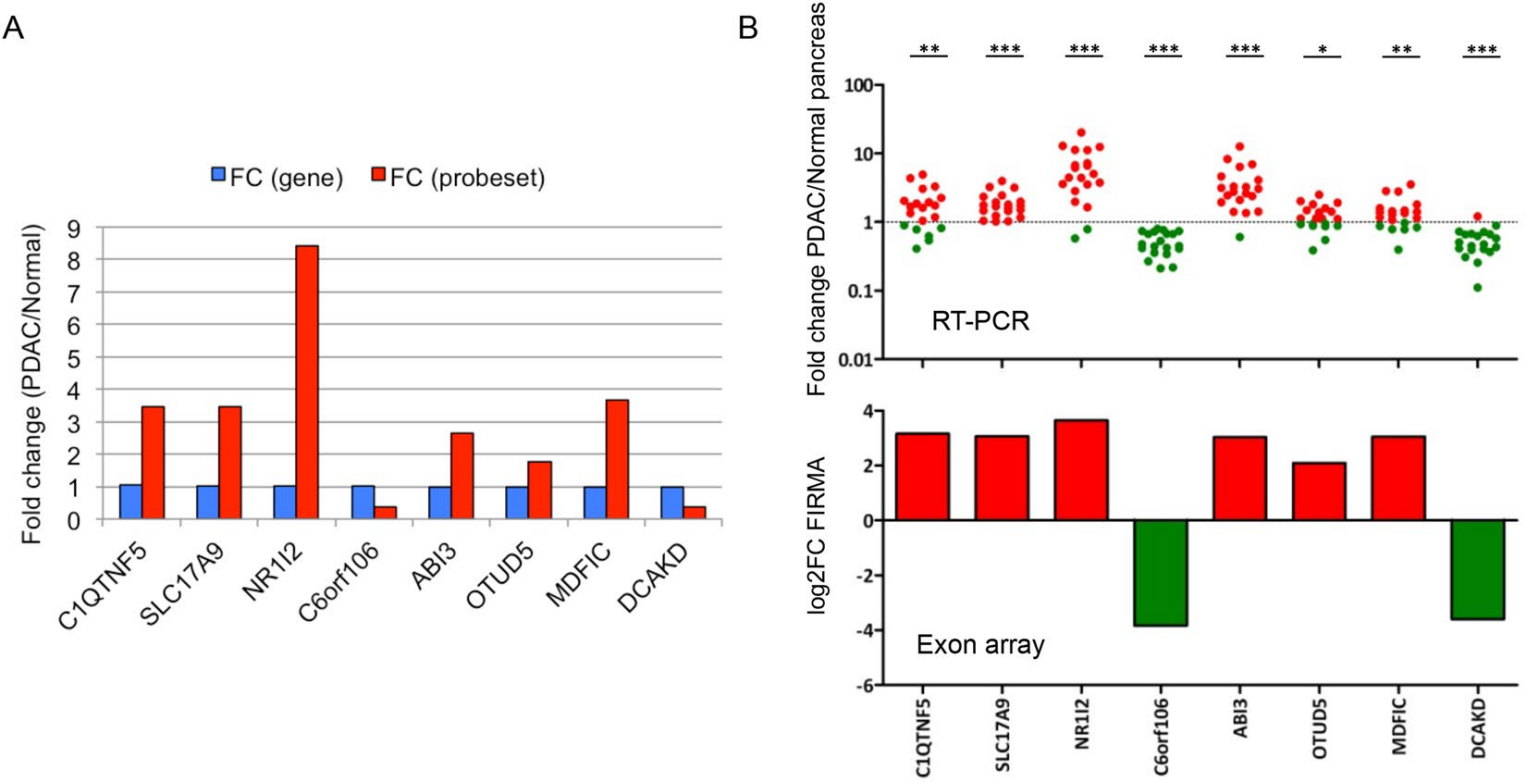
Real-time PCR validation of exon array data. (**A**) Selection of 8 splicing probe-sets from non-differentially expressed genes. For these 8 genes/probe-sets, there were no differences in the level of gene expression, but significant differences in the level of probe-set expression. (**B**) RT-PCR results of the 8 probe-sets. RT-PCR results are represented as scatter dot plots of expression fold change in PDAC compared to normal pancreas, with the significance level shown at the top (* < 0.05, ** < 0.01, *** < 0.001). Corresponding exon array results are reported in histograms of log_2_ FIRMA fold change (PDAC/normal) values. The two sets of results corresponded well with each other very well.

Real-time PCR was performed using 20 PDAC and six normal pancreas tissues for eight splicing events from randomly selected non-differentially expressed genes (fold change between 0.9–1.1): *C1QTNF5*, *SLC17A9*, *NR1I2*, *C6orf106*, *ABI3*, *OTUD5*, *MDFIC* and *DCAKD* (Fig. 3A **)**. As presented in Fig. 3B, the PCR results validated the exon array data with significantly higher levels for the included exons (*C1QTNF5*, *SLC17A9*, *NR1I2*, *ABI3*, *OTUD5*, *MDFIC*) and significantly lower levels for the two skipped exons (*C6orf106* and *DCAKD*) in PDAC samples compared to normal pancreas. Thus, these data validated the accuracy of FIRMA prediction for exon skip/inclusion.

### Identification of cell surface DE and AS regulated genes as candidate biomarkers and therapeutic targets

**Figure 4:**
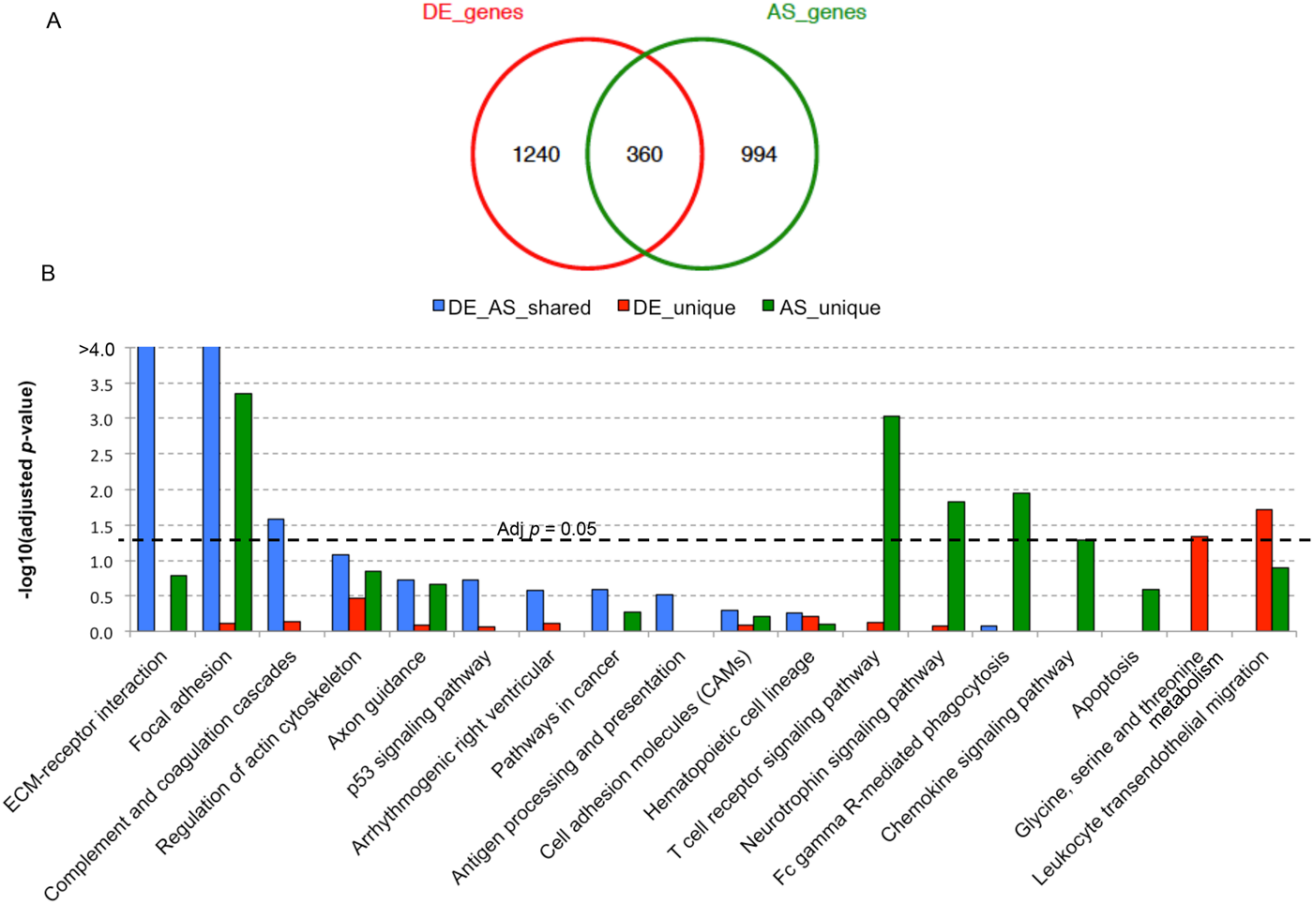
Comparison between DE and AS genes. (**A**) The overlapping patterns between differentially expressed (DE) genes and alternatively spliced (AS) genes. (**B**) The KEGG pathway enrichment for unique DE genes (red), unique AS genes (green) and genes with both DE and AS signatures (blue). The –log_10_ (adjusted *p*-values) is shown at the y-axis.

We next established which genes were commonly regulated by both differential expression (DE) and AS, by comparing the 1,354 AS to the 1,600 DE protein-coding genes (Fig. 4, Supplementary Table S3A). 360 genes showed both DE and AS signatures in PDAC, accounting for 22.5% and 26.6% of total DE and AS genes, respectively (Fig. 4A). Upon assessing the pathways that these 360 genes modulate, we found a significant enrichment for ECM-receptor interaction (adj. *p* = 9.78e-11) and focal adhesion (adj. *p* = 9.05e-06), while immunity-related pathways (particularly T cell receptor signalling (adj. *p* = 9.29e-04), Fc gamma receptor-mediated phagocytosis (adj. *p* = 1.12e-02), chemokine signalling, as well as neurotrophin signalling (which supports survival, development and function of neurons) (all with adj. *p* < 0.05) were predominantly affected by AS (Fig. 4B, Supplementary Table S3B). Actin cytoskeleton and axon guidance genes appeared to be regulated by both DE and AS, as well as unique AS events. This was also largely supported by IPA (Supplementary Table S3C).

AS and DE events that affect cell surface proteins might represent an exciting opportunity for their use as biomarkers for detection and/or as therapeutic targets. A total of 135 cell surface protein-coding genes that are regulated at both gene expression and alternative splicing level are listed in Supplementary Table S3D.

**Figure 5:**
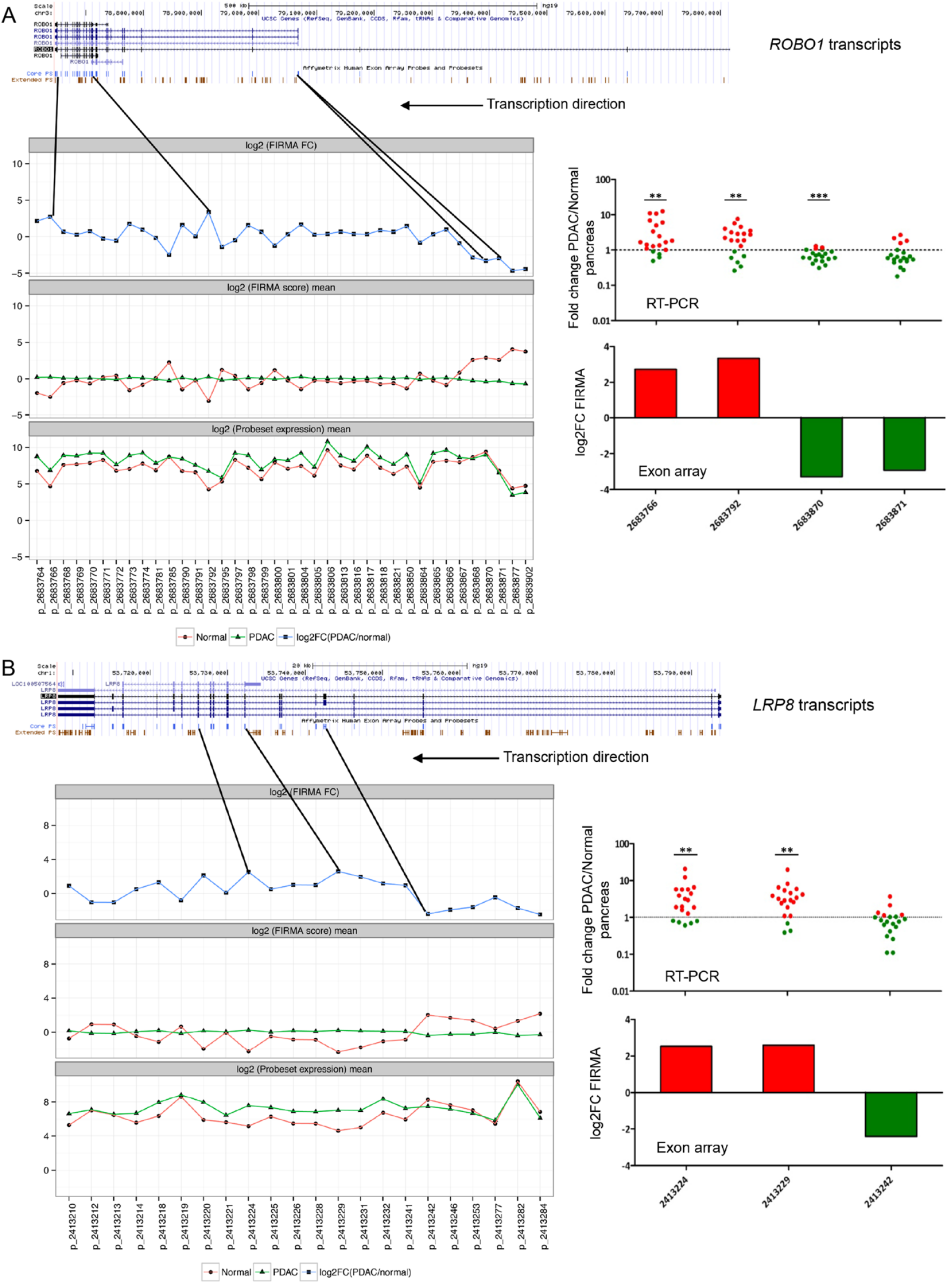
Analysis of multiple splicing events for *ROBO1* and *LRP8*, and validation with RT-PCR. (**A**) Alternative splicing for *ROBO1*. (**B**) Alternative splicing for *LRP8*. The UCSC transcripts are shown at the top. For all quantified core probe-sets, means for probe-set expression and FIRMA values (log_2_ scaled), and the log_2_ FIRMA fold changes (PDAC/normal) were shown underneath UCSC transcripts. The probe-sets chosen for RT-PCR validation were shown with the black solid lines, with their positions within the transcripts indicated. RT-PCR results for the chosen probe-sets are represented as scatter dot plots of expression fold change in PDAC compared to normal pancreas, with the significance level shown at the top (* < 0.05, ** < 0.01, *** < 0.001). Corresponding exon array results are also reported in histograms of log_2_ FIRMA fold change (PDAC/normal) values. Both *ROBO1* and *LRP8* are coding for cell-surface proteins, and both were transcribed in the reverse strand.

The splicing pattern of two genes encoding the cell surface proteins, *ROBO1* (an axon guidance gene) and *LRP8* (Low Density Lipoprotein Receptor-Related Protein 8, which plays a critical role in the migration of neurons during development), were assessed by real-time PCR (Fig. 5). *ROBO1* and *LRP8* displayed multiple splicing events according to FIRMA prediction (Supplementary Fig. S4) and were upregulated in PDAC compared to normal tissues, with a fold change of 2.03 for *ROBO1* and 1.68 for *LRP8*. Real-time PCR validated the exon array results for the selected splicing events in both *ROBO1* (Fig. 5A) and *LRP8* (Fig. 5B). Of note, while a lower expression of probe-sets 2683871 and 2413242 was seen by RT-PCR, the values did not reach statistical significance. For *ROBO1*, a higher skipping rate for probe-sets/exons at the 5′ end (represented by probe-set 2683870 and 2683871), but a higher inclusion rate for probe-sets/exons at the 3′ end (e.g., 2683766) and for some cassette exons in the middle (e.g., 2683792) were seen in PDAC compared to normal (Fig. 5A). Similarly for *LRP8*, probe-sets/exons at the 5′ end (e.g., probe-set 2413284) and cassette exons close to the 5′ side (represented by 2413242) appeared to be skipped, but exons in the middle (e.g., 2413224 and 2413229) and those close to the 3′ end tended to be included in PDAC compared to normal (Fig. 5B **)**. Thus, it is likely that different promoters were utilised and shorter transcripts become the primary choice in PDAC.

In addition to surface proteins affected by DE and AS being candidate drug targets, the discovery of PDAC-associated splice variants might also represent a novel diagnostic opportunity. Using mass spectrometry, Menon *et al*. have detected a number of protein products of gene splice variants in the plasma of a KrasG12D/Ink4a/Arf mouse model of PDAC^20^. When these splice isoforms were compared to our AS signatures, 32 common AS genes were found; interestingly, these genes were highly enriched for focal adhesion (adj. *p* = 1.24e-03) and ECM-receptor interaction (adj. *p* = 2.49e-03), including *TNC*, several collagen genes, *ZYX*, *MYLK* and *FN1*. These highly conserved AS variant proteins represent an intriguing lead for further validation of their diagnostic potential.

### Hypersensitive genes

We next tested the behaviour of putative driver genes recently reported by Bailey *et al*.^17^ regarding differential expression and alternative splicing events. Fifty-six mutated genes were reported using the IntoGen software (*Q* < 0.1, Supplementary Table S4A from ref. 17); of these, four genes showed significant DE signatures between PDAC and normal in our dataset (based on the double threshold of adjusted *p*-value < 1e-04 and absolute FC > 2), including three upregulated: *KRAS*, *TGFBR1* and *CALD1*, and one downregulated (*FBLN2*) gene in PDAC. An additional eight DE genes could be identified (using just adjusted *p*-value < 1e-04), including four upregulated (*MACF1*, *TNIK*, *TP53BP2* and *PBRM1)*, and four downregulated genes: *ACVR1B*, *RBM10*, *BCORL1* and *TTC18* in PDAC compared to normal (Supplementary Table S4B).

When the AS patterns were investigated for these 56 genes, seven genes displayed AS signatures. Among them, *TGFBR2*, *KDM6A*, *CALD1* and *MYCBP2* all had highly skipped probe-sets, while *TGFBR1*, *MACF1* and *ITPR3* possessed both highly skipped and included probe-sets in PDAC compared to normal (Supplementary Table S4C **)**. Thus, a set of three significantly mutated genes that were significantly mutated and also showed both DE and AS was derived, including *TGFBR1*, *CALD1* and *MACF1* (Supplementary Fig. S5). Interestingly, both alternative first and last exons were significant events for *TGFBR1*, implying the differential selection of regulatory elements in PDAC development for this gene (Supplementary Fig. S5C **)**.

We further expanded our analysis to genes that were significantly targeted by deletions and amplifications, revealed by the copy number GISTIC analysis^17^. A total of 17 out of 21 genes (81.0%) in significantly deleted regions that had strong DE signatures were downregulated in PDAC based on our double threshold, demonstrating good concordance between gene expression and copy number changes, including *ARHGDIG*, *RPL3L*, *SNORA64*, *RAB26*, *GOLGA8A/B* and *MAPK8IP2* (Supplementary Table S4D **)**. The 121 genes within the significantly amplified regions in PDAC were also significantly differentially expressed, with 74 (61.2%) upregulated genes, including *ITGA2*, *VCAN*, *THBS2*, *IL7R*, *LOX* and *EDIL3* (Supplementary Table S4E **)**.

We then inspected the AS patterns of genes within deleted and amplified regions, and found that 12 deleted and 95 amplified genes also displayed strong AS (Supplementary Table S4F and G **)**. Of these, 34 genes additionally showed DE signatures, consisting of *ABCA3*, a deleted and downregulated gene in PDAC, and 33 amplified and upregulated genes (including *ADAM19*, *CAST*, *CD180*, *CSTB*, *DOCK2*, *F2R*, *MX1*, *PCDH7*, *RUNX1*, *TGFBI* and *VCAN)* are listed in Supplementary Table S4H.

The inclusion of the three significantly mutated genes (*TGFBR1*, *CALD1* and *MACF1*) to the latter 34 genes resulted in 37 genes that we refer to as a set of ‘hypersensitive’ genes, as they are frequently targeted by somatic mutations, copy number aberrations, differential expression and alternative splicing, indicating their increased intrinsic susceptibility to multiple genomic aberrations in PDAC.

### Spliceosomal and splicing regulator genes in PDAC

**Figure 6:**
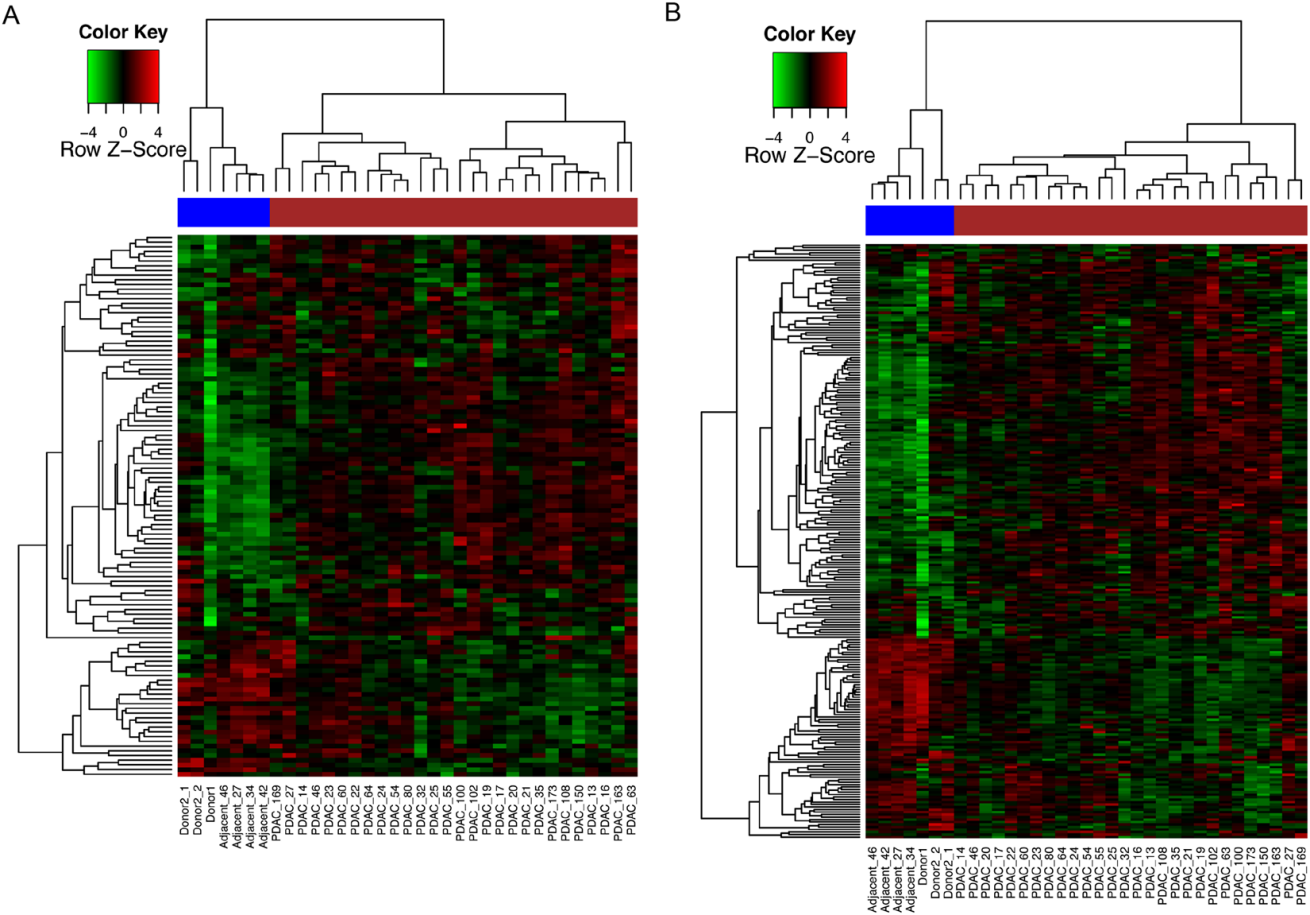
Heatmap showing the expression profiles of 119 (**A**) and 238 (**B**) spliceosomal genes, with the upregulated genes shown in red and downregulated in green. PDAC samples were indicated with the brown bar, while normal samples were indicated with the blue bar.

Finally, in order to understand the behaviour of the spliceosomal machinery in PDAC, we have assessed the DE and AS of genes involved in splicing. Among the 140 spliceosomal genes reported by Zhou *et al*.^21^, the expression data for 119 (85%) were investigated in our study (Supplementary Table S5A). Of these, 28 (23.5%) genes were significantly differentially expressed (using an adjusted *p*-value threshold of 1e-04) in PDAC, with 22 upregulated genes, the top two being *PRPF40A* and *SNRNP27* (Supplementary Table S5A). Interestingly, unsupervised hierarchical clustering of the 119 spliceosomal genes showed clear separation between normal and PDAC samples as illustrated in Fig. 6A. To detect a potential influence of age on PDAC clustering, we investigated the association between PDAC sample clustering of spliceosomal genes and patient age, but found no significant difference in age between the two groups of patients (Wilcoxon test, *p* = 0.90). This was also the case when we explored the association between age and patients’ clustering based on significant AS probe-sets (Fig. 2A), Wilcoxon test, *p* = 0.20.

Previously, Carrigan *et al*. analysed AS in PDAC cell lines and demonstrated that 28 of 92 (~30%) spliceosomal genes had significantly decreased expression in pancreatic cancer compared with normal pancreas^11^; in our study, expression profiles for 18 of these 28 genes were identified and only three of them (*SART1*, *CIRBP* and *HTATSF1*) were significantly downregulated in PDAC (adjusted *p* < 0.05), while nine (*DNAJC8*, *DHX9*, *DHX8*, *SLU7*, *RSRC2*, *MBNL2*, *NCBP2*, *PRPF4B* and *MBNL3*) were significantly upregulated (adjusted *p* < 0.05) (Supplementary Fig. S6 **)**. This difference is likely due to the different analytical platforms and sample types utilised in the two studies. When the selection of spliceosomal genes was expanded to all 238 splicing regulators based on the Ensembl annotation (Supplementary Table S5B), 73/238 (30.7%) of these genes were found to be differentially expressed (adjusted *p*-value < 1e-04), with 39 upregulated and 34 downregualted genes in PDAC relative to normal. The top upregulated genes (FC > 2) included *PRPF40A*, *SNRNP27, LGALS3*, and *ADAR*, and the top downregulated genes (FC < 0.5) included *APOBEC2*, *CELF3*, *ADARB2* and *CSDC2* (Supplementary Table S5B). Unsupervised clustering based on all 238 spliceosomal genes was again able to differentiate PDAC from the healthy pancreas (Fig. 6B **)**.

Interestingly, 12 spliceosomal genes: *AQR*, *C19orf43*, *DHX16*, *PPM1G*, *PUF60*, *SNRPD3*, *SRSF1*, *SRSF4*, *TCERG1*, *U2AF2*, *USP39* and *WTAP* also showed the evidence of AS - all had skipped exon signatures in PDAC (Supplementary Table S2). Thus, genes in spliceosomal machinery are themselves regulated by both DE and to a lesser extent by AS. This could potentially explain and be one of the reasons for a compromised splicing fidelity in cancer. Of note, we did not find any association between the aberrations of spliceosomal machinery and patients’ age.

## Discussion

Here, we provide the first comprehensive tissue-based AS landscape of PDAC, with the report of a number of newly identified AS events. We show that the most common alterations in the protein-coding AS genes in PDAC are skipped exon and alternative 1^st^ exon, followed by intron retention. Exon skipping was previously highlighted as being the most common AS form involved in shaping eukaryotic evolution^22^, and we show here that the same mechanism is also utilised in PDAC development. In contrast, intron retention, a rare event in normal eukaryotic tissues (most commonly seen in plants, fungi and protozoa)^1^ seemed to be also hijacked in PDAC evolution. This inclusion of intronic sequences within the mRNA (sometimes referred to as the phenomenon of exonization) has been reported in cancer^23^, suggesting that aberrant AS is not merely a side effect of cancer, but a *bona fide* regulator of cancer development^22^.

In addition to our validation, a number of genes affected by AS described here have already been reported, providing an independent validation of our data. A total of 121 genes (8.9%) found to have AS signatures in our PDAC cohort were also found to have skipped exons across different tissues^4^ (Supplementary Table S6). The AS of *CALD1*, *COL6A3*, *FN1*, *MAST2*, *LGR5* and *ITGB4* were previously validated in colon cancer using RT-PCR^24^, while *CLSTN1*, *AUP1*, *CTNND1*, *CALD1* and *COL6A3* were shown to exhibit tumour-specific splice variants in colon, bladder and prostate cancers^8^. *ADAM12*, *DKK3*, *GSN*, *TNFSF11*, *CDH3*, *CXCL5*, *HCK*, *ITGA5* and *VEGFC* have also been shown to possess known or novel splice variants in lung cancer^25^. The *BIRC5* (survivin) gene and its spliced isoforms have been shown to be associated with prostate cancer cell proliferation and aggressive phenotypes^26^; and tumour-associated splice variants of *MACF1*, *ITGB3*, *TLE3*, *SHC1*, *ETS1* and *BCAS1* have been reported in lung, prostate, breast cancer and glioblastoma^27–32^.

In PDAC, AS in *PSMD2*, *PTPN18*, *SUPT16H*, *CUL4A*, *NIN*, *SLK*, and *ABCC3* were also reported previously^11^, and increased AS of the *KLF6* tumour suppressor gene was shown to correlate with prognosis and tumour grade^33^. Interestingly, in our data, four other members of the Krüppel-like family, *KLF5*, *KLF7*, *KLF12* and *KLF16*, also showed significant AS events (Supplementary Table S2A). The AS in human tissue factor (*TF* or *F3*) was shown to promote tumour growth in an orthotopic pancreatic cancer model^34, 35^, and our data also show a highly skipped event for this gene (within the first exon) in PDAC (probe-set: 2423935; FIRMA log2FC = −4.69, adj. *p* = 1.99e-08).

Overall, collagen genes showed the highest versatility in AS in PDAC; interestingly, a switch in alternate promoters in collagen IX during fracture healing^36^, and a dynamic process of tumour-specific AS in several exons of *COL6A3* were previously reported^37^. While in evolutionary terms increased splicing presents novel opportunities for expansion of gene families, its functional implications are far-reaching, particularly in PDAC, where the abundant stroma, predominantly composed of collagens, is a characteristic and pronounced feature. It is highly plausible that alternatively spliced collagen genes play a critical role in intense stromal remodelling with important repercussions to mechanical and stiffness properties, shown recently to be implicated in progression and invasive properties of PDAC^38^.

Of note, while our samples were pre-selected to predominantly comprise tumour cells, it is possible that a large number of AS signatures seen here in collagen and other ECM genes are derived from the desmoplastic stromal compartment still present in our samples. Therefore, future studies that involve the microdissection and AS profiling of both tumour and stromal cells will further enrich our understanding of this phenomenon, and inform on tumour and stroma specific AS events in PDAC. Furthermore, to compare the AS/DE profile of chronic pancreatitis to that of normal and/or tumour tissues would also be an important further step in understanding the degree to which differences described here are tumour-specific.

Around a third of the significant genes in our study were shown to be affected by both DE and AS (Fig. 4), and they were particularly enriched for ECM-receptor interaction and focal adhesion pathways as well as complement and coagulation cascades. Of these genes, 135 encode cell surface proteins. Although it still remains to be fully established if these events are PDAC-specific, such surface proteins might represent an as yet untapped source of both potential imaging tools or drugable targets. Furthermore, the discovery of PDAC-associated splice variants in body fluids might present a novel diagnostic opportunity, as at least 32 AS gene products were found as circulating proteins in mouse plasma^20^. These represent a rich pool of non-invasive diagnostic candidates that now need to be explored and validated directly in human biofluids.

Interestingly, we describe here a group of hypersensitive genes, which showed predilection to be affected by mutations, copy number aberrations, DE and AS signatures. While the detailed mechanistic annotation for such a multidimensional regulation of this set of genes remains to be further established, their functionality in the ECM-receptor interactions, focal adhesion, collagen fibril organisation and actin cytoskeleton provides a versatility of options in communication networks between cancer cells and their ever-changing microenvironment.

Finally, we also looked at the spliceosomal machinery itself. A deregulation of around a third of genes belonging to this dynamic ribonucleoprotein complex was found, with upregulation being more prevalent than downregulation; this has also been established for the majority of other cancers. Based on the percentage of upregulated *vs*. downregulated splicing factor genes, PDAC appeared to be more similar to renal cell carcinoma and lung adenocarcinoma than to prostate, colorectal and breast cancers^5^. Of note, the spliceosomal signature itself was sufficient to clearly separate the normal and cancer specimens, suggesting its cancer-specificity; curiously, we also show that several spliceosomal genes can undergo alternative splicing themselves.

It was previously shown that spliced isoforms follow the principle of parsimony and adopt the simplest structural folds, with the most pronounced changes seen in the exposed surface of the affected proteins^39^. Integration of our data with the RNA-Seq data and development and refinement of the computational prediction methods to inform on the resultant protein sequence and corresponding 3D structural models on a global scale is now needed to gain full benefit from the available AS data.

In summary, we provide the most comprehensive landscape of AS events in PDAC to date, with underlying changes in the spliceosome and its regulators. We also report a group of alternatively spliced genes that encode surface and circulating proteins. These represent novel candidates of translational relevance as potential diagnostic and therapeutic targets in pancreatic adenocarcinoma.

## Materials and Methods

### Samples and RNA isolation

A total of 43 freshly-frozen pancreatic samples (37 PDAC, four histologically normal samples adjacent to cancer and two normal donor tissue samples) were analysed in this study. Samples were obtained from the Department of Pathology, Verona, Italy, after informed consent with full ethical approval from the Institutional Review Boards of The University of Verona. The experiment and methods were conducted in accordance with the Declaration of Helsinki. The demographic and clinical information of the patients and samples are summarised in Table 1. Of note, the samples have been selected based on tumour cellularity, so over 80% of PDAC specimens had >60% cancer cell content.

Total RNA was isolated using TRIzol (Invitrogen); 1.5 μg of total RNA was further processed (depleted of ribosomal RNA and labelled) according to supplied protocols (Affymetrix, Santa Clara, CA, USA).

### Affymetrix Exon array expression analysis

Affymetrix GeneChip® Human Exon 1.0 ST Arrays, comprising ~1.4 million probe-sets consisting of over 5 million individual probes and >300,000 transcript clusters (group of probe-sets targeting individual exons in genes and noncoding transcripts) were used for gene-level expression profiling and AS analysis. Labelling using Affymetrix GeneChip Whole Transcript (WT) ST Labeling Assay and subsequent hybridization were performed according to the manufacturer’s instructions. After scanning, CEL files were checked for quality and analysed following the pipeline described in Rodrigo-Domingo *et al*.^40^. Exon array data files have been submitted to Gene Expression Omnibus (GEO) under the accession number of GSE63158, a SuperSeries consisting of the gene-level data GSE56560 and the exon-level data GSE63111.

Within the pipeline, the “aroma. affymetrix” R package^41^ was used for data preprocessing, normalisation and summarisation to produce transcript, probe-set and probe-level intensities. Here, only the “core” probe-sets, supported by the most reliable evidence from RefSeq and full-length mRNA GenBank records containing complete coding DNA sequence (CDS) information and transcript clusters were used. This was followed by intensity filtering across samples as recommended by the Affymetrix White Paper^10^. Data obtained from one replicate of the two donor samples were of low quality and were subsequently removed. Using the limma R package^42^, transcript cluster expression intensities were further analysed to identify differentially expressed (DE) transcripts between PDAC and control groups. The raw *p*-values were adjusted using the Benjamini-Hochberg (BH) model^43^. Differentially expressed transcript clusters were identified using a double threshold of adjusted *p*-value < 1e-04 and absolute fold change (FC) > 2. The transcript clusters were further matched to Ensembl genes and gene symbols.

### Alternative splicing (AS) analysis

Differential alternative splicing events were detected using FIRMA (Finding Isoforms using Robust Multichip Analysis)^44^. FIRMA scores for all filtered probe-sets were calculated using “aroma. affymetrix”. After log_2_ transformation, the differential splicing analysis was conducted using limma. Alternatively spliced probe-sets were identified using a double threshold of adjusted *p*-value < 1e-06 and absolute log_2_ FC > 2. The ANalysis Of Splice VAriation (ANOSVA) method^45^ was also used as an additional filter with an adjusted *p*-value < 1e-06 at both the probe and probe-set levels. Only protein-coding genes were finally selected according to the Ensembl gene annotation. Identified AS events in PDAC were categorised into common patterns of alternatively spliced exonic regions according to the Ensembl alternative splicing event set^4, 46^, *i.e*. alternative 5′ and 3′ sites, alternative first and last exon, skipped and consecutive exon, intron retention and isoform and mutually exclusive exons.

### Gene set enrichment tests

The gene set enrichment tests were performed using the Database for Annotation, Visualization and Integrated Discovery (DAVID)^12^ to inspect overrepresented Gene Ontology (GO) Biological Process terms and KEGG pathways. The *p*-values were adjusted using the BH model. The functional and pathway analyses were also conducted using Ingenuity Pathway Analysis (IPA, Ingenuity® Systems, www.ingenuity.com). To identify surface protein coding genes, the GO Cellular Component terms for genes were explored using DAVID to determine those coding for plasma membrane proteins.

### Real-time PCR validation

First strand cDNA was prepared from 1 µg of total human pancreatic RNA using Quantitect Reverse Transcription kit (Qiagen, Crawley, UK). Real-time PCR was performed on a 7500 Real Time PCR System (Applied Biosystems, Warrington, UK) using SYBR Green dye (Qiagen) according to the manufacturer’s instruction. Specific primers were designed and evaluated for amplification efficiency with the use of Universal Human Reference RNA (Agilent technologies, Stockport, UK) (Supplementary Table S7). To confirm the exon array data, relative changes of expression were shown for each target after normalization to the reference genes *HPRT1*, *RPLP0* and *S16*, according to the formula: 2ΔΔCt^47^. In addition to samples used for Affymetrix experiments, eight more PDAC and four more pancreatic normal tissues were used; in total, the validation was performed on 20 PDAC and six normal samples.

### Molecular subtype analysis

In addition to our exon array dataset, an analogous dataset from Zhang *et al*.^15^, was also used for clinical inferences. The two data sets (gene expression and clinical follow-up) were compiled, processed and merged as previously reported^48^. To identify PDAC molecular subtypes using the Collisson gene signature^14^, non-negative matrix factorisation (NMF) consensus clustering^49^ was employed to identify stable sample clusters based on normalised or z-score standardised expression values for each dataset. The R package ‘ConsensusClusterPlus”^50^ was also used to verify sample clustering. After sample clustering and grouping, Kaplan-Meier (KM) analysis and Log Rank test as well as Cox proportional hazards model were undertaken for survival analyses examining the impacts of grouping on overall survival using the R ‘survival’ package (https://cran.r-project.org/web/packages/survival/).

## Electronic supplementary material

Supplementary Figures

Supplementary Table S1

Supplementary Table S2

Supplementary Table S3

Supplementary Table S4

Supplementary Table S5

Supplementary Table S6

Supplementary Table S7
