# Supplementary material for: Splice variants as novel targets in pancreatic ductal adenocarcinoma
Source: Sci Rep. 2017 Jun 7;7:2980. doi: 10.1038/s41598-017-03354-z (PMC5462735; doi:10.1038/s41598-017-03354-z)
Supplement: Supplementary file 1 — Supplementary Figures [file 41598_2017_3354_MOESM1_ESM.pdf]

Splice variants as novel targets in pancreatic ductal adenocarcinoma

Jun Wang[1], Laurent Dumartin[1], Andrea Mafficini[2], Pinar Ulug[1], Ajanthah Sangaralingam[1], Namaa Audi Alamiry[1], Tomasz P Radon[1], Roberto Salvia[2], Rita T Lawlor[2], Nicholas R Lemoine[1], Aldo Scarpa[2], Claude Chelala[1,#] and Tatjana Crnogorac-Jurcevic[1,#]

[1] Centre for Molecular Oncology, Barts Cancer Institute, Queen Mary University of London, London, UK;
[2] ARC-Net Research Centre and Department of Diagnostics and Publich Health, Section of Pathology, University and Hospital Trust of Verona, Verona, Italy
[#]These authors jointly supervised this work
Correspondence and requests for materials should be addressed to J.W. (email: j.a.wang@qmul.ac.uk) or T.C.J. (email: t.c.jurcevic@qmul.ac.uk)

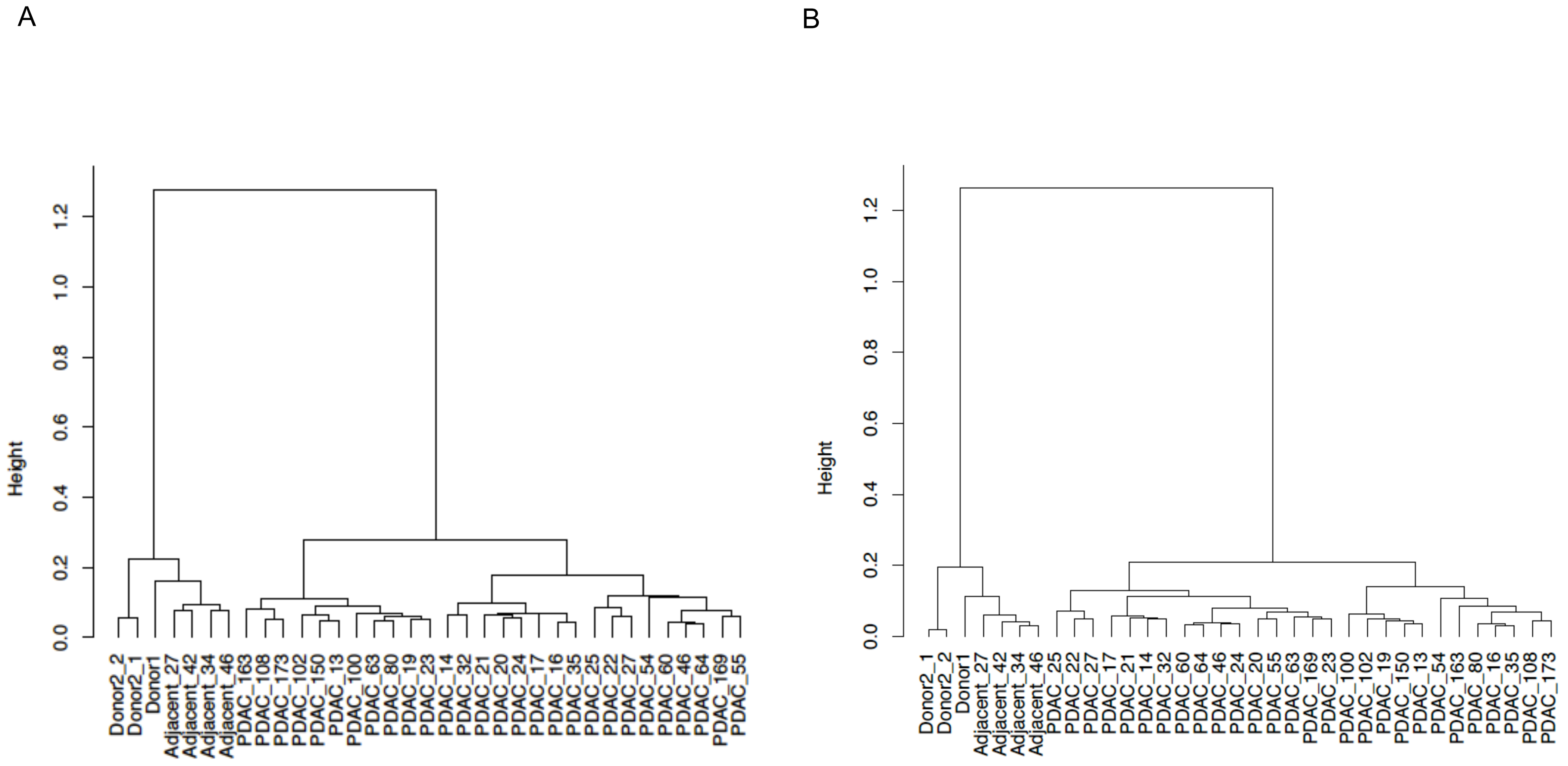

Figure S1. Unsupervised hierarchical clustering based on probeset (A) and transcript (B) expression level

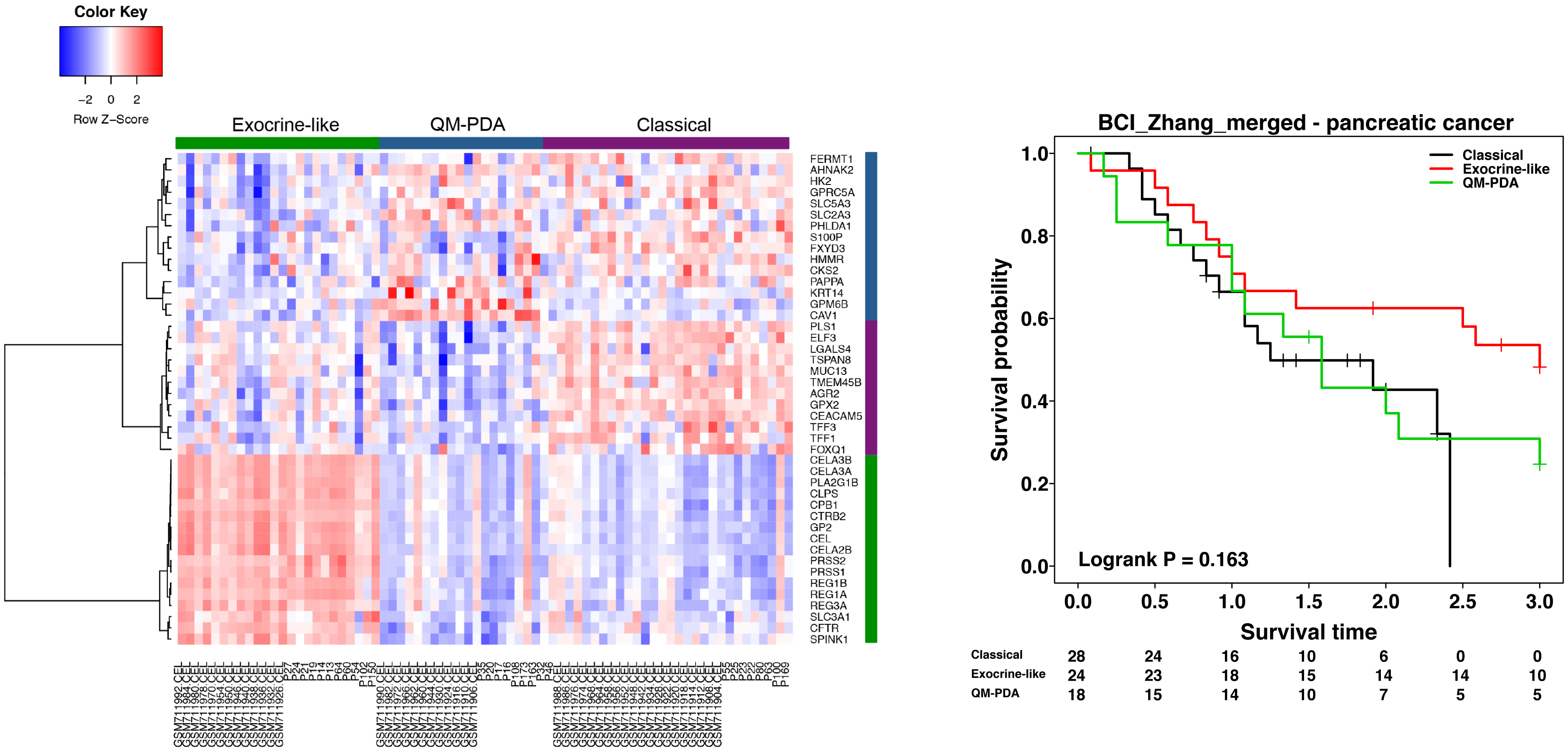

Figure S2. Gene expression and sample clustering of Collisson signature (62 genes) in BCI and Zhang merged dataset

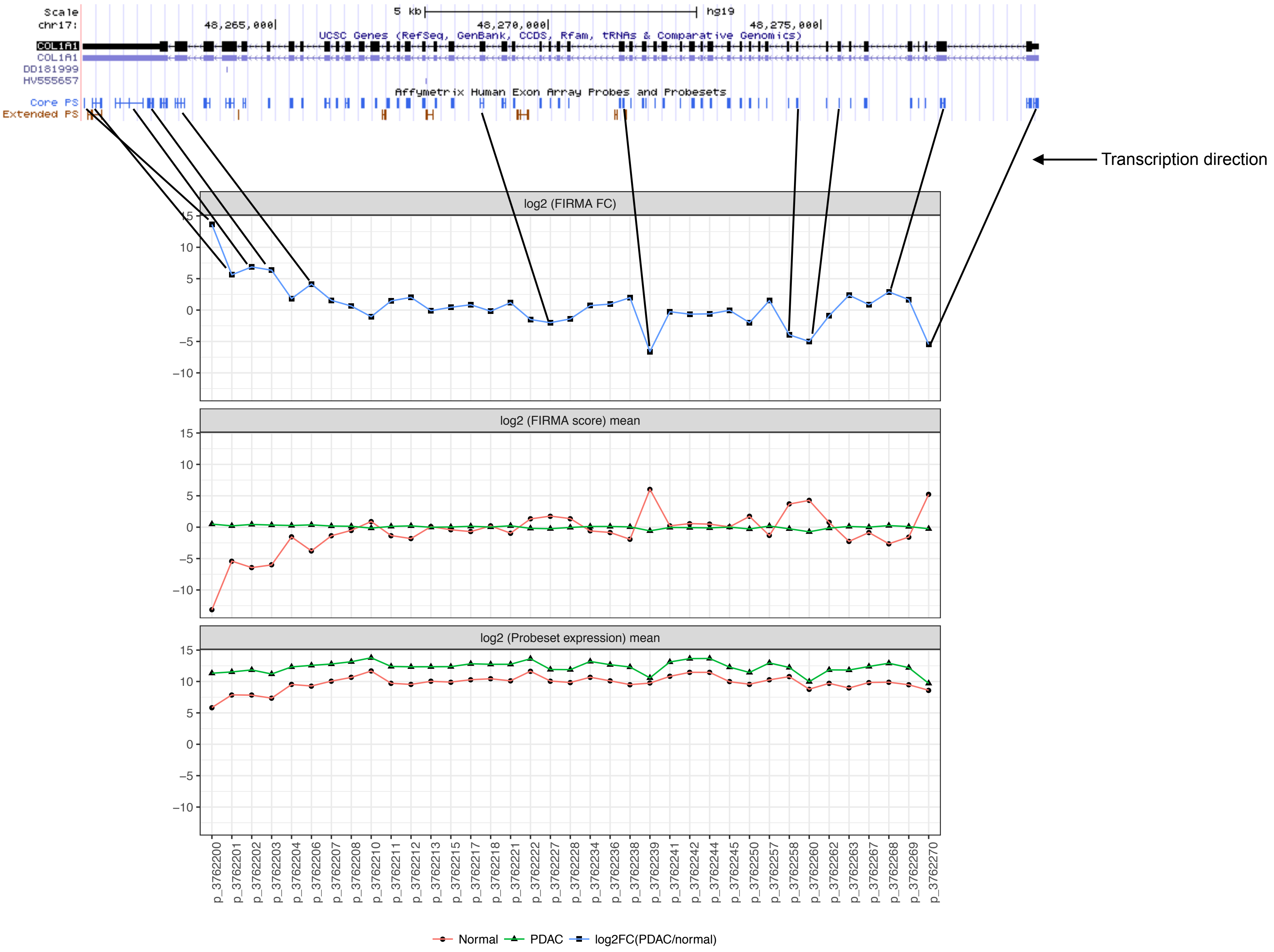

Figure S3A. alternative splicing in COL1A1

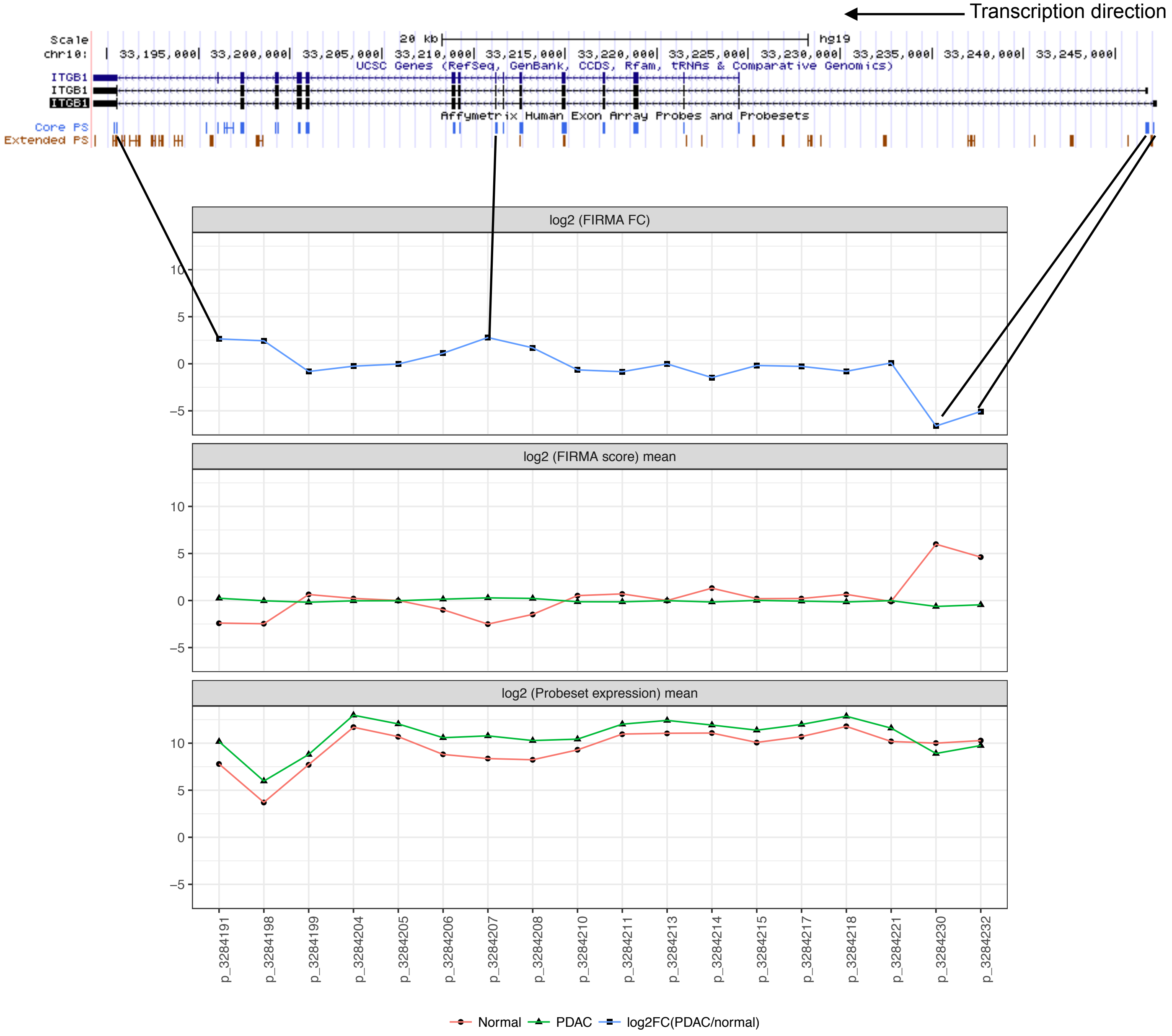

Figure S3B. alternative splicing in ITGB1

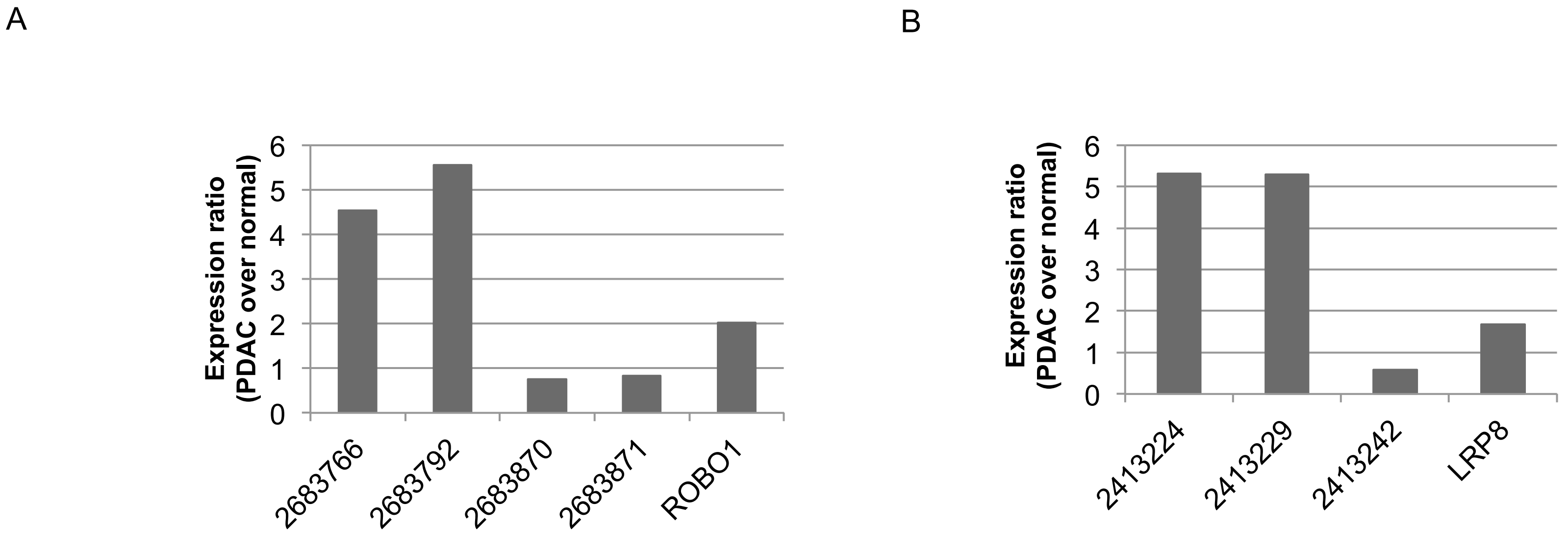

Figure S4. The expression ratio (PDAC over normal) of AS probesets and gene for ROBO1 and LRP8

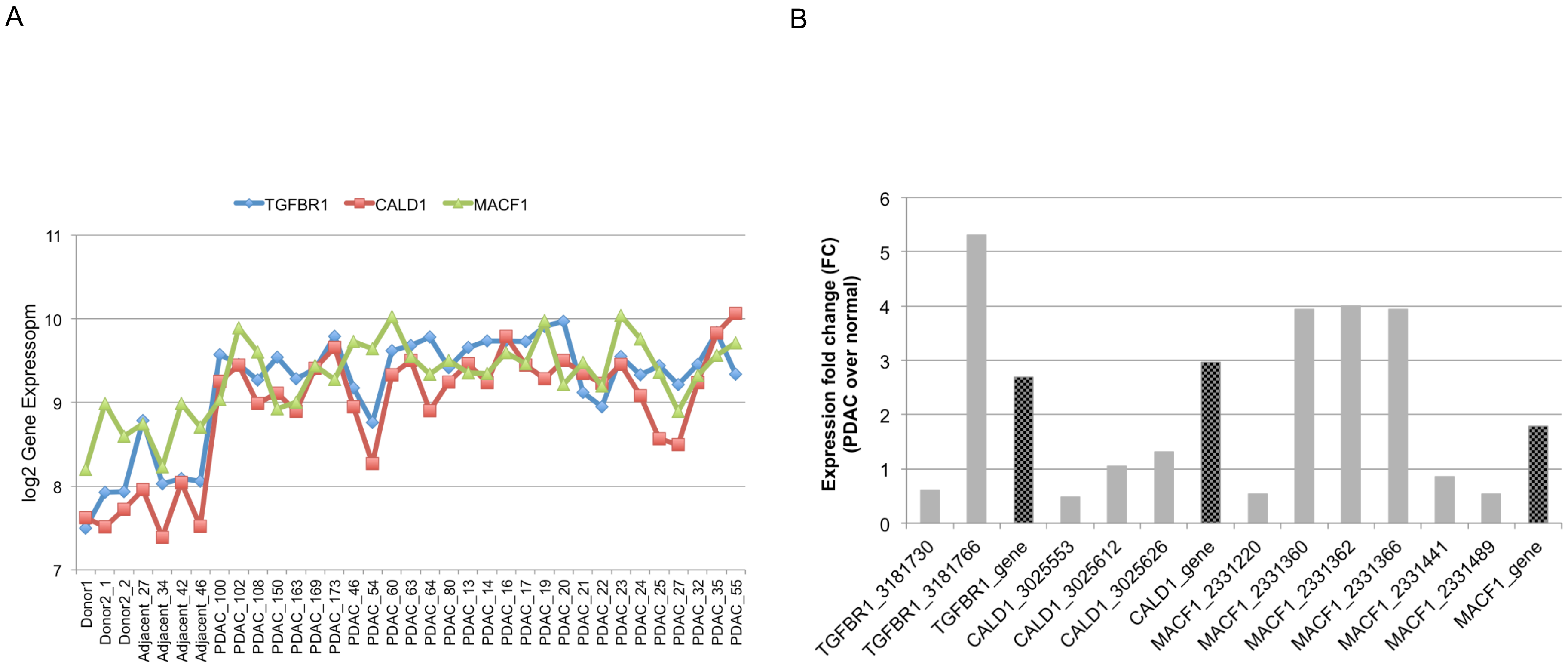

Figure S5A/B. Expression fold changes for TGFBR1, CALD1 and MACF1 in gene and probeset/exon levels

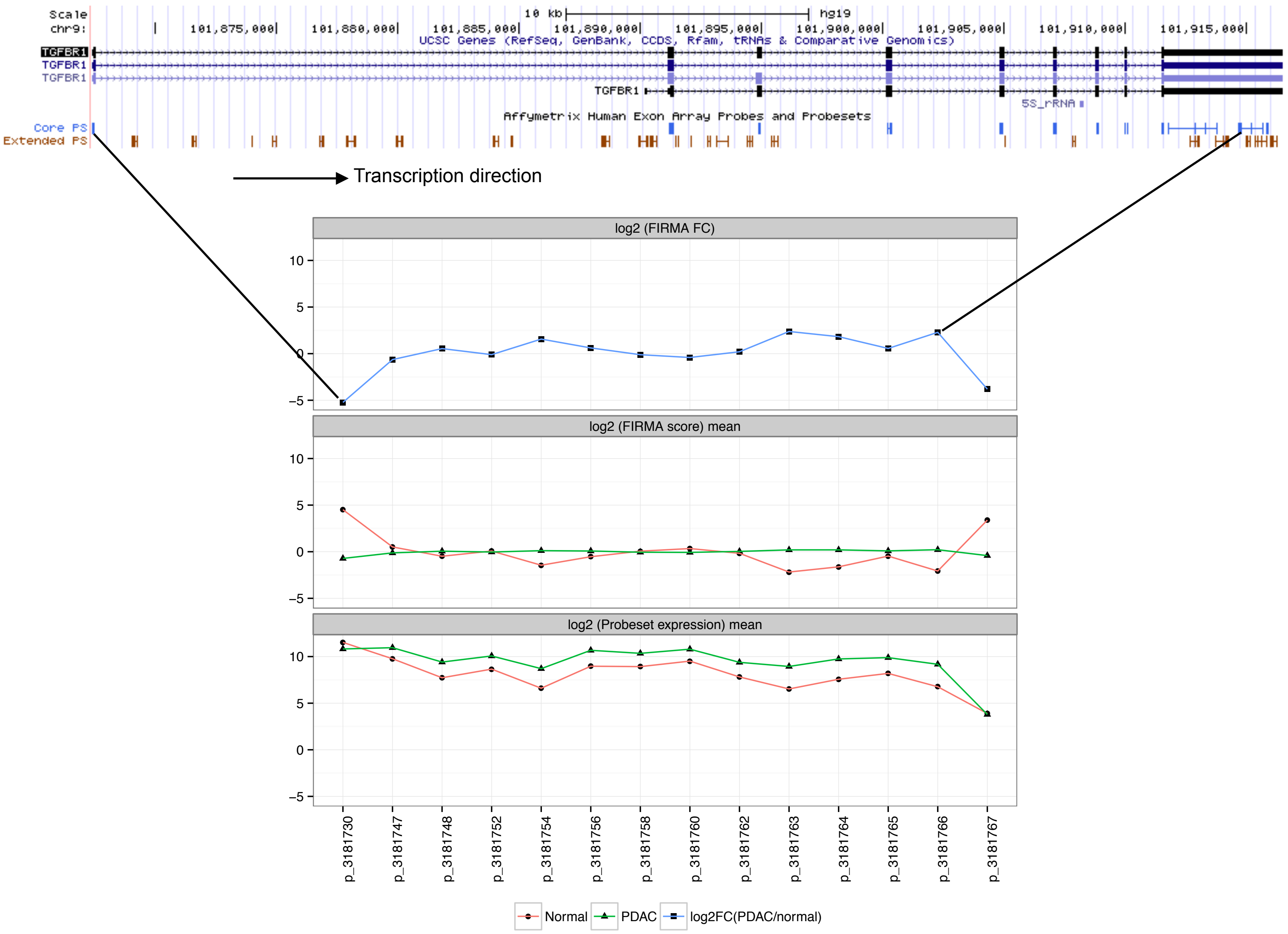

Figure S5C. Alternative splicing in TGFBR1

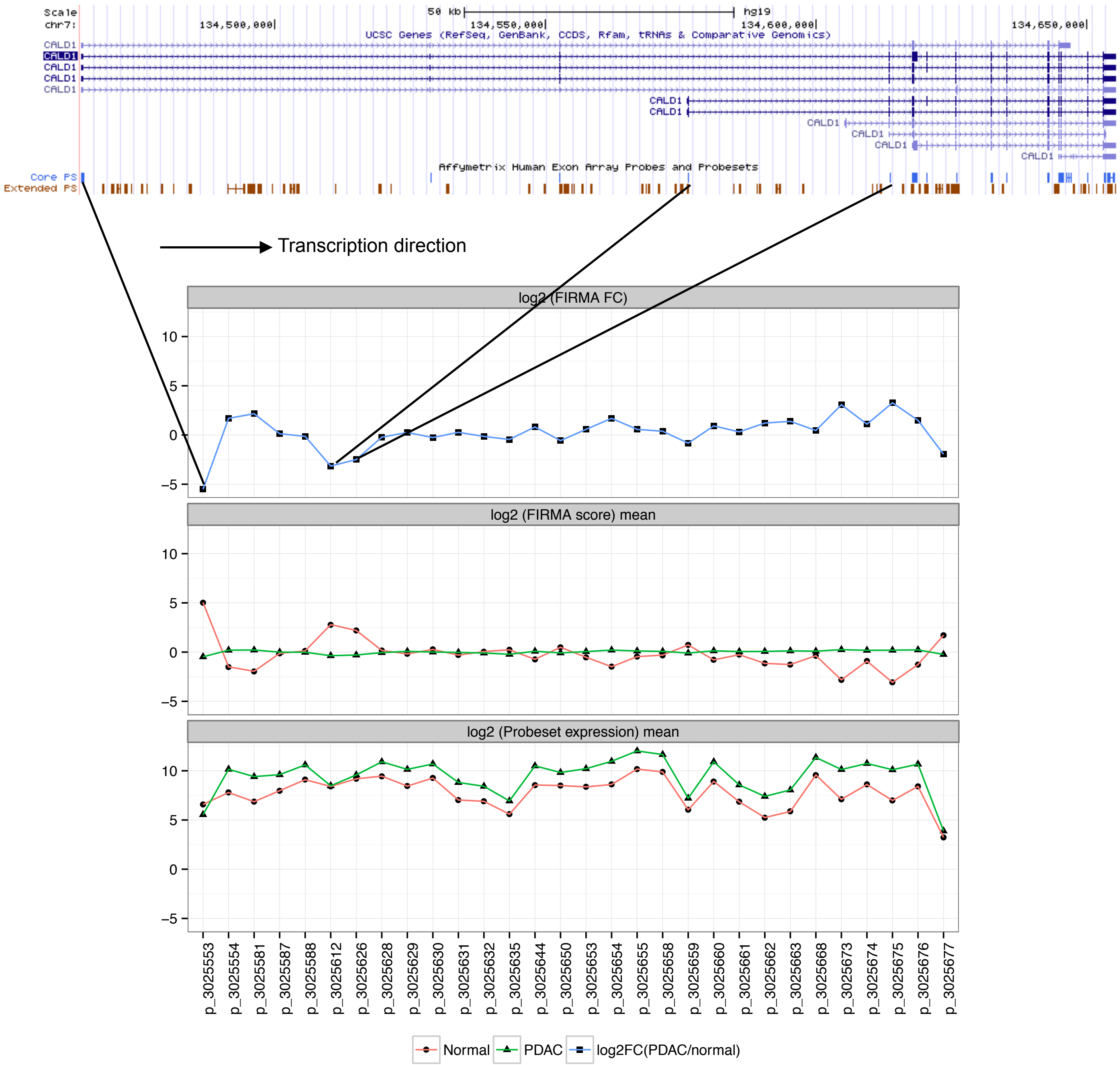

Figure S5D. Alternative splicing in CALD1

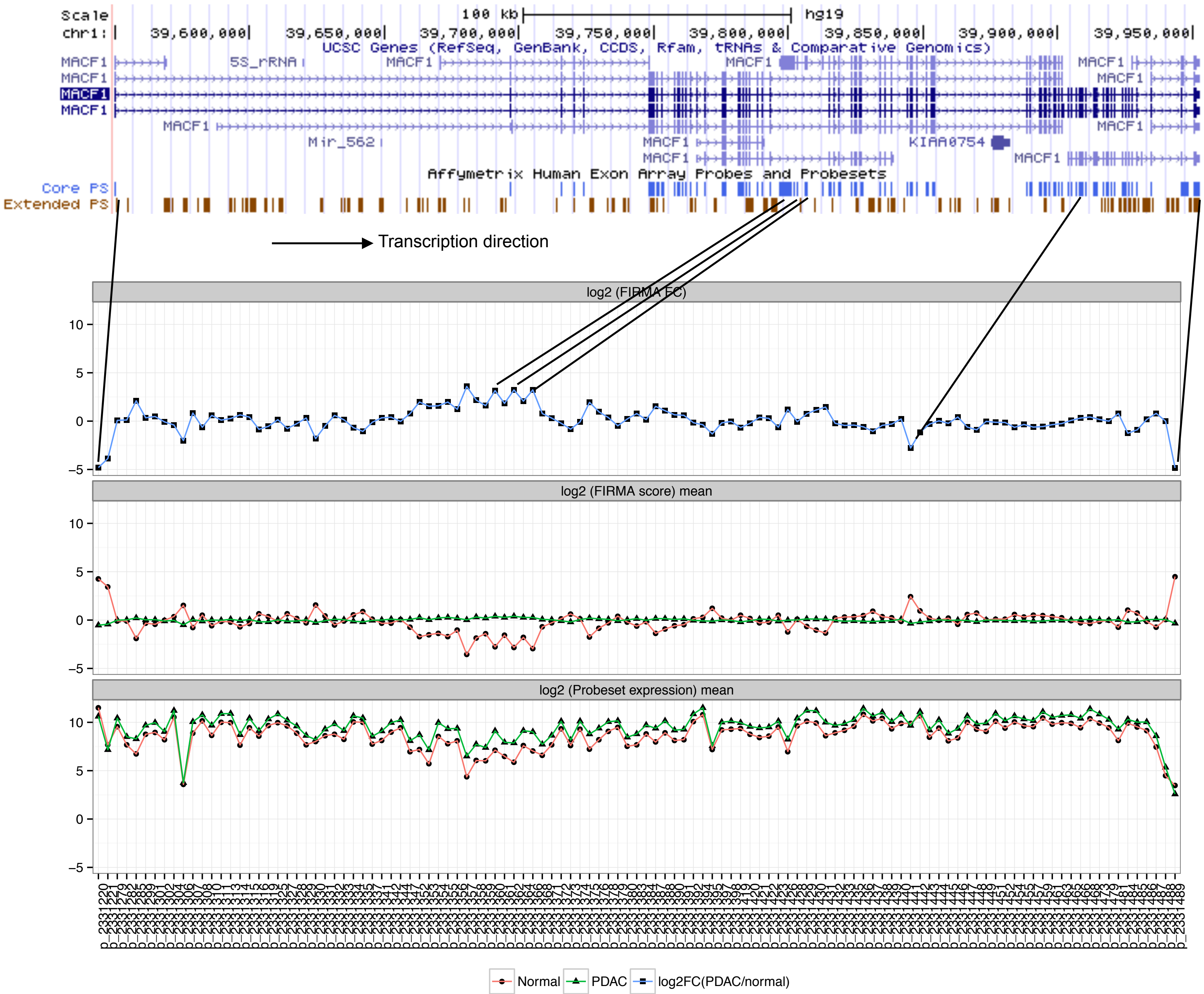

Figure S5E. Alternative splicing in MACF1

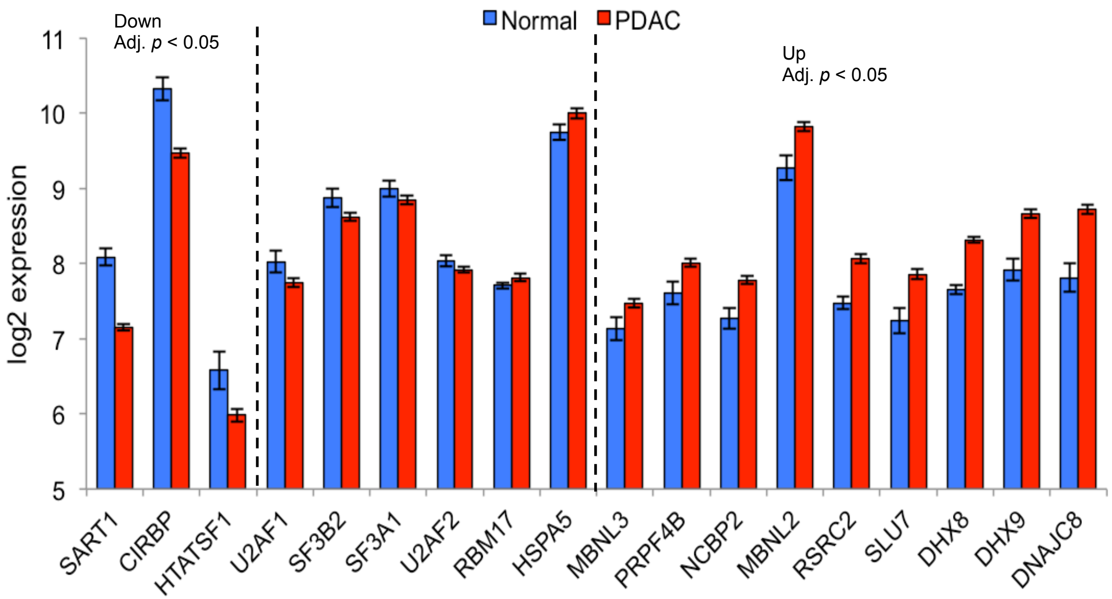

Figure S6. The DE signatures for previously reported spliceosomal genes
